# Correlations, Information Backflow, and Objectivity in a Class of Pure Dephasing Models

**DOI:** 10.3390/e24020304

**Published:** 2022-02-21

**Authors:** Nina Megier, Andrea Smirne, Steve Campbell, Bassano Vacchini

**Affiliations:** 1 Dipartimento di Fisica “Aldo Pontremoli”, Università degli Studi di Milano, Via Celoria 16, 20133 Milan, Italy; andrea.smirne@unimi.it (A.S.); bassano.vacchini@mi.infn.it (B.V.); 2 Istituto Nazionale di Fisica Nucleare, Sezione di Milano, Via Celoria 16, 20133 Milan, Italy; 3 International Centre for Theory of Quantum Technologies (ICTQT), University of Gdansk, 80-308 Gdansk, Poland; 4 School of Physics, University College Dublin, Belfield, D04 Dublin, Ireland; 5 Centre for Quantum Engineering, Science, and Technology, University College Dublin, Belfield, D04 Dublin, Ireland

**Keywords:** non-Markovianity, quantum Darwinism, dephasing, correlations, Jensen–Shannon divergence

## Abstract

We critically examine the role that correlations established between a system and fragments of its environment play in characterising the ensuing dynamics. We employ a dephasing model with different initial conditions, where the state of the initial environment represents a tunable degree of freedom that qualitatively and quantitatively affects the correlation profiles, but nevertheless results in the same reduced dynamics for the system. We apply recently developed tools for the characterisation of non-Markovianity to carefully assess the role that correlations, as quantified by the (quantum) Jensen–Shannon divergence and relative entropy, as well as changes in the environmental state, play in whether the conditions for classical objectivity within the quantum Darwinism paradigm are met. We demonstrate that for precisely the same non-Markovian reduced dynamics of the system arising from different microscopic models, some exhibit quantum Darwinistic features, while others show that no meaningful notion of classical objectivity is present. Furthermore, our results highlight that the non-Markovian nature of an environment does not a priori prevent a system from redundantly proliferating relevant information, but rather it is the system’s ability to establish the requisite correlations that is the crucial factor in the manifestation of classical objectivity.

## 1. Introduction

The necessity for effective means to describe how a quantum system interacts with its surrounding environment has precipitated a burgeoning area of research. In many instances, one is solely focused on the dynamics of the system of interest, and therefore, environmental effects can be phenomenologically modelled, rendering the complex system dynamics tractable [1,2]. While highly effective, such an approach neglects to account for the root cause of the ensuing dynamics of the system. Reverting to a full microscopic description, where the system and environment interact and evolve according to an overall unitary dynamics, reveals that the correlations established between the system and the environment during their interaction play an important role in the resulting open dynamics of the system [1,2]. These correlations are the basis for notions of classical objectivity [3,4,5,6,7] and are also known to play a key role in the characterisation of the dynamics, in particular, if the system undergoes a Markovian (memoryless) or non-Markovian evolution [8,9]. Both notions of classical objectivity and non-Markovian evolution have been the object of experimental investigations; see, for example, [10,11,12,13] and [14,15,16,17,18,19,20], respectively.

However, a given open system dynamics does not arise from a unique microscopic system–environment model, and rather, there are infinitely many system–environment models that result in the same system evolution [21]. Such an insight calls for a more careful analysis of the information exchanges between the system and its environment, allowing to more precisely pin down the relevant contributions which give rise to, for example, Markovian vs. non-Markovian dynamics [22], or establish the conditions for classical objectivity [23,24,25]. This becomes particularly subtle since under such a microscopic picture, the environment is typically composed of many constituent subsystems, and therefore it is relevant to assess the complementary role that global correlations established between the system and the whole environment play compared to correlations shared between the system and smaller environmental fragments. With regards to the former, it was recently demonstrated that without the creation of strong global correlations in the form of entanglement, reasonable conditions for objectivity are not satisfied [24,25], while for the latter, it appears that only the correlations shared between the system and a small subset of the environmental degrees of freedom are relevant for the characterisation of the system dynamics [22,26].

In this work, we attempt to unravel the contribution that various correlations play in the characterisation of an open system dynamics. To that end, we consider a spin-star dephasing model, where several different initial environmental states, which in turn lead to significantly different correlation profiles, nevertheless produce the same reduced dynamics for the system [21]. We employ recently developed tools for understanding the emergence of non-Markovianity in terms of the correlations established between the system and environment, as well as changes in the environmental state [22,27], to put into evidence the quite different role that these features play when characterising the dynamics, either in terms of its non-Markovian character or its ability to establish the conditions necessary for classical objectivity. We show that two different microscopic descriptions of the evolution that lead to the same reduced dynamics of the system can exhibit significant differences with regards to classical objectivity within the quantum Darwinism framework. Our work therefore demonstrates that the non-Markovian character of an evolution does not necessarily affect a system’s ability to redundantly proliferate information to the environment, thus contributing to the ongoing efforts to unravel their relation [28,29,30,31] or possible lack thereof [32,33]; note, in particular, the recent analysis in [34] complementary to ours.

The remainder of the paper is organised as follows. In Section 2, we introduce the spin-star model with different initial conditions that is our focus. Section 3 introduces the correlation measures that are our key figures of merit and examines how they spread in the dependence on the initial condition. We analyse various information fluxes in Section 4 and their dual role characterising the non-Markovian nature of the dynamics and the redundant spreading of relevant system information to environmental constituents. Finally, we draw our conclusions in Section 5.

## 2. Dephasing Models

Let us introduce the models for which we want to study the role of correlations in determining important features of the overall and reduced dynamics. We recall that in being interested in the reduced dynamics of the system in a system–environment setting, the full specification of a model includes the choice of the initial environmental state. We therefore consider a set of *N* two-level systems with frequency $\omega_{E}$, interacting with a two-level system with frequency $\omega_{S}$, via the microscopic Hamiltonian

(1) $$\begin{array}{ccc} H & = & \frac{\hbar \omega_{S}}{2}\sigma_{z}⊗1_{2^{N}}+\sum_{k=1}^{N}g_{k}\sigma_{z}⊗\sigma_{z}^{k}+\sum_{k=1}^{N}\frac{\hbar \omega_{E}}{2}1_{2}⊗\sigma_{z}^{k}. \end{array}$$

With $\sigma_{z}^{k}$, we denote the operator $1_{2^{\left(k-1\right)}}⊗\sigma_{z}⊗1_{2^{\left(N-k\right)}}$, where the Pauli matrix $\sigma_{z}$ acts on the *k*-th environmental qubit, while $1_{d}$ indicates the identity operator in a space of dimension *d*, and the $g_{k}$’s are the system environment coupling constants. Such an interaction corresponds to a so-called spin-star setting, in which a central spin is coupled to neighbouring environmental degrees of freedom, which can be described by a collection of non-interacting spins. In particular, the considered coupling term is such that it only affects the coherences of the system since $\sigma_{z}⊗1_{2^{N}}$ is a constant of motion, thus describing a dephasing dynamics. We investigate the time evolution of these degrees of freedom in the hypothesis of the existence of a closed reduced dynamics for the central spin system, that is to say, assuming the initial overall state factorised according to $\rho_{SE}\left(0\right)=\rho_{S}\left(0\right)⊗\rho_{E}\left(0\right)$. We consider models in which the initial environmental state is given by a tensor product of identical states, namely

(2) $$\begin{array}{ccc} \rho_{E}\left(0\right) & = & \overset{N}{\underset{k=1}{⨂}}\varrho_{E}, \end{array}$$

with

(3) $$\begin{array}{ccc} \varrho_{E} & = & \left(\begin{array}{cc} p & c\\ c & 1-p \end{array}\right), \end{array}$$

where p∈[0,1] and without loss of generality, we can take *c* real in the range $\left|c\right|⩽\sqrt{p(1-p)}$. This initial environmental state allows us to explore not only the total correlations, but also their establishment as a function of the fraction of environmental degrees of freedom we are taking into consideration.

Starting from the fact that the total unitary evolution operator in the interaction picture can be written in the form

(4) $$\begin{array}{ccc} U(s) & = & \sum_{\left\{m_{k}\right\}}e^{-i\sigma_{z}\left(\sum_{k=1}^{N}g_{k}m_{k}\right)s}⊗|\left\{m_{k}\right\}\rangle \langle \left\{m_{k}\right\}|, \end{array}$$

where the vectors $\{|\left\{m_{k}\right\}\rangle =|m_{1}\rangle ⊗\ldots ⊗|m_{N}\rangle \}$, such that $\sigma_{z}^{k}|m_{k}\rangle =m_{k}|m_{k}\rangle$ with $m_{k}\in \left\{-1,1\right\}$, denote the basis of eigenvectors of the operator ${⨂}_{k=1}^{N}\sigma_{z}^{k}$ in the environmental space, we obtain for the evolved state of system and environment the expression

(5) $$\begin{array}{ccc} \rho_{SE}\left(s\right) & = & \left(\begin{array}{cc} \rho_{11}\left(0\right)\overset{N}{\underset{k=1}{⨂}}\rho_{k}\left(s\right) & \rho_{10}\left(0\right)\overset{N}{\underset{k=1}{⨂}}\sigma_{k}\left(s\right)\\ \rho_{01}\left(0\right)\overset{N}{\underset{k=1}{⨂}}\sigma_{k}^{*}\left(s\right) & \rho_{00}\left(0\right)\overset{N}{\underset{k=1}{⨂}}\rho_{k}^{*}\left(s\right) \end{array}\right), \end{array}$$

where

(6) $$\begin{array}{ccc} \rho_{k}\left(s\right) & = & \left(\begin{array}{cc} p & ce^{-i2g_{k}s}\\ ce^{i2g_{k}s} & 1-p \end{array}\right) \end{array}$$

and

(7) $$\begin{array}{ccc} \sigma_{k}\left(s\right) & = & \left(\begin{array}{cc} pe^{-i2g_{k}s} & c\\ c & \left(1-p\right)e^{i2g_{k}s} \end{array}\right). \end{array}$$

An important feature of the considered class of evolutions appears when considering the associated reduced dynamics. Indeed, taking the partial trace with respect to the environmental degrees of freedom, one immediately obtains

(8) $$\begin{array}{ccc} \rho_{S}\left(s\right) & = & \left(\begin{array}{cc} \rho_{11}\left(0\right) & \rho_{10}\left(0\right)\chi \left(s\right)\\ \rho_{01}\left(0\right)\chi^{*}\left(s\right) & \rho_{00}\left(0\right) \end{array}\right) \end{array}$$

with

(9) $$\begin{array}{ccc} \chi (s) & = & \prod_{k=1}^{N}\left[\cos\left(2g_{k}s\right)-i\left(2p-1\right)\sin\left(2g_{k}s\right)\right], \end{array}$$

where we have used the identity

(10) $$\begin{array}{c} \sum_{\left\{m_{k}\right\}}e^{-i2\left(\sum_{k=1}^{N}g_{k}m_{k}\right)s}\langle \left\{m_{k}\right\}|\rho_{E}\left(0\right)|\left\{m_{k}\right\}\rangle =\prod_{k=1}^{N}\left[\cos\left(2g_{k}s\right)-i{\langle \sigma_{z}^{k}\rangle }_{\varrho_{E}}\sin\left(2g_{k}s\right)\right], \end{array}$$

and ${\langle \ldots \rangle }_{\varrho_{E}}$ denotes the expectation value with respect to the state $\varrho_{E}$ given in Equation (3), so that the reduced dynamics is exactly the same for all initial environmental states with the same diagonal matrix elements. Therefore, we have a whole class of dephasing models, parametrised by the coherence, *c*, of the environmental state given by Equation (3), leading to exactly the same reduced dynamics. The existence of different environments equally affecting a given system has been studied in different contexts, with the purpose of allowing for more convenient numerical treatments [35,36,37,38]. The occurrence of the very same reduced evolution starting from different microscopic dynamics in a controlled setting was recently considered also in [21], in order to investigate the physical mechanism behind memory effects in quantum dynamics.

For the sake of simplicity, and in order to allow for analytical results, we consider the case in which all coupling constants are taken to be equal to a reference value *g*, so that all environmental units evolve in the same way throughout the dynamics, as well as a uniform distribution of the populations in the initial environmental components, namely p=1/2. In particular, we address situations in which $\varrho_{E}$ in Equation (3) ranges from pure, for c=±12, to maximally mixed for c=0. The maximally mixed state corresponds in particular to a situation in which the reduced environmental state is unaffected by the interaction with the system. This choice has the advantage of providing a simple parameter characterizing the different considered initial conditions, while, as can be inferred from [33,39], the results are expected to be robust with respect to noise in the initial preparation.

We now consider possible physical manifestations of the difference in the microscopic dynamics and related correlations studying the onset of Darwinistic behaviour and non-Markovianity in various environmental scenarios.

## 3. Spreading of Correlations

Let us first study the establishment and spread of correlations in the considered scenarios. As discussed in detail in many publications [6,23,24,25,40,41,42,43,44], this feature might have an impact on the notion of objectivity for a quantum state, in the spirit of so-called quantum Darwinism [3] (see, for example, ref. [7] for a recent review and references therein). We see that it also provides us with interesting insights in the study of quantum non-Markovianity [8,9].

**Figure 1:**
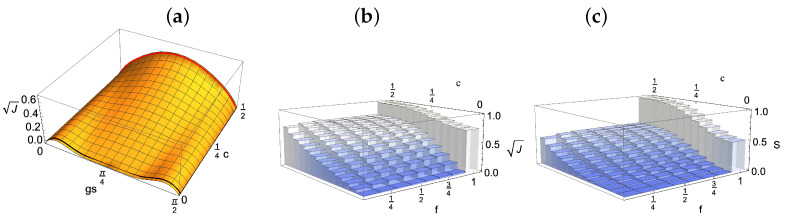
(**a**) Amount of correlations shared between the system initially in the plus state $|+\rangle =\left(1/\sqrt{2}\right)(|0\rangle +\left|1\rangle \right)$ and one of the environmental qubits, evaluated by considering the QJSD12, comparing this bipartite state with the product of its marginal as a function of time (in inverse units of the coupling parameter) and of the value *c* of initial coherence in the environmental states. The quantity is renormalised to the value corresponding to a maximally entangled state. Here and in the following figures, the environment is composed of N=8 units. The black and red lines correspond to c=0 and c=12. (**b**) Distance between total state and product of its marginals at the reference time gs=π/4 as quantified by the QJSD12, expressed as a function of the fraction of considered environmental qubits and of the value, *c*, of coherences in the environmental states. The total state includes the system and a fraction, f, of the environmental qubits. (**c**) The same quantity obtained considering as the quantifier the relative entropy, thus recovering the mutual information, still keeping the normalisation to the value corresponding to the maximally entangled state. In both figures, we see the emergence of a plateau for c=12, which is gradually washed out for smaller values of *c*, namely when moving from a model in which the environmental units have coherences to a fully diagonal state.

As is clear from Equations (8) and (9), for a uniform coupling, the reduced dynamics has a period of π/2 in the variable gs, so that we will consider times up to π/(2g). In particular, the system is fully decohered for gs=π/4. This decoherence is connected to the establishment of correlations with the environmental qubits; however, as shown in Figure 1, these correlations (as quantified by the quantum Jensen–Shannon divergence defined in the following subsection) are greater the more environmental qubits we take into account. In particular, for c=0, the reduced system is only correlated at this point of time with the environment as a whole. The overall state according to Equation (5) then reads

(11) $$\rho_{SE}\left(s\right)=\frac{1}{2^{N}}(\begin{array}{cc} \rho_{11}\left(0\right)\overset{N}{\underset{k=1}{⨂}}\left(\begin{array}{cc} 1 & ce^{-i2gs}\\ ce^{i2gs} & 1 \end{array}\right)\; & \rho_{10}\left(0\right)\overset{N}{\underset{k=1}{⨂}}\left(\begin{array}{cc} e^{-i2gs} & c\\ c & e^{i2gs} \end{array}\right)\\ \rho_{01}\left(0\right)\overset{N}{\underset{k=1}{⨂}}\left(\begin{array}{cc} e^{i2gs} & c\\ c\; & e^{-i2gs} \end{array}\right) & \;\rho_{00}\left(0\right)\overset{N}{\underset{k=1}{⨂}}\left(\begin{array}{cc} 1 & ce^{i2gs}\\ ce^{-i2gs}\; & 1 \end{array}\right) \end{array}).$$

Given that we are considering a dephasing dynamics, a natural choice for the initial condition for the system is a pure state of the form $\rho_{S}\left(0\right)=\left|+\rangle \langle +\right|$, with $|+\rangle =\left(1/\sqrt{2}\right)(|1\rangle +\left|0\rangle \right)$, exhibiting the maximum amount of coherence so that ρij(0)=12 for i,j=0,1. Starting from this expression one can consider marginals in which less and less environmental units are involved. In particular, we will denote as $\rho_{SE_{fN}}$ the state obtained by tracing over all environmental units not contained in a fraction f of the environment. For the extreme cases f=0 and f=1, we recover the reduced and total states, respectively.

### 3.1. Quantifiers of Correlations

In order to understand the spreading of correlations in the different models, we are, therefore, interested in their dependence on the considered fraction. In general, given a distinguishability quantifier between quantum states, say *D*, which is a quantity defined on pairs of quantum states such that D(ρ,σ)⩾0 with equality if the states coincide, one can use it as a quantifier of correlations in a bipartite state considering the expression $D(\rho_{SE},\rho_{S}⊗\rho_{E})$. For the sake of this study, we consider the square root of the quantum Jensen–Shannon divergence (QJSD12) and the relative entropy. We use both as quantifiers of bipartite correlations by renormalizing to the value assumed for the case of a maximally entangled state. The choice of the QJSD12 is motivated by its use in the framework of non-Markovianity [45,46], while the relative entropy is typically used in the framework of quantum Darwinism [7] due to its connection with the mutual information.

The QJSD12 is defined in terms of the Jensen–Shannon divergence [47] according to

(12) $$\begin{array}{ccc} \sqrt{J(\rho ,\sigma )} & = & \sqrt{S\left(\frac{\rho +\sigma }{2}\right)-\frac{1}{2}S\left(\rho \right)-\frac{1}{2}S\left(\sigma \right)}, \end{array}$$

where S(ρ)=−Trρlogρ denotes the von Neumann entropy and logarithms are considered in base 2. This quantity, besides being a well-known distinguishability quantifier, was recently shown to be a distance [48,49]. In particular, when used to evaluate correlations, it takes the form

$$\begin{array}{c} \sqrt{J(\rho_{SE},\rho_{S}⊗\rho_{E})}=\sqrt{S\left(\frac{\rho_{SE}+\rho_{S}⊗\rho_{E}}{2}\right)-\frac{1}{2}S\left(\rho_{SE}\right)-\frac{1}{2}S\left(\rho_{S}\right)-\frac{1}{2}S\left(\rho_{E}\right)}, \end{array}$$

taking the value $\sqrt{2-(5/8)\log5}\approx 0.74$ when $\rho_{SE}$ corresponds to the maximally entangled state in $C^{2}⊗C^{2N}$. We denote as $\sqrt{J}$ the quantity rescaled by this factor, thus assuming unity for maximally entangled states.

The relative entropy is defined according to [47]

(13) $$\begin{array}{ccc} S(\rho ,\sigma ) & = & \mathrm{Tr}\rho \log\rho -\mathrm{Tr}\rho \log\sigma , \end{array}$$

so that when used to quantify correlations, it leads to the mutual information

(14) $$\begin{array}{ccc} S(\rho_{SE},\rho_{S}⊗\rho_{E}) & = & S\left(\rho_{S}\right)+S\left(\rho_{E}\right)-S\left(\rho_{SE}\right), \end{array}$$

providing a natural quantifier of both classical and quantum correlations. Considering again logarithms in base 2, it takes the value 2 for the maximally entangled state in $C^{2}⊗C^{2N}$, so we denote as S the quantity rescaled by a factor 2.

### 3.2. Model Dependence of Correlation Formation

The key quantities to be considered in the study of the establishment of correlations between the system and different parts of the environment in the different considered models are, therefore, $\sqrt{J(\rho_{SE_{fN}},\rho_{S}⊗\rho_{E_{fN}})}$ and $S(\rho_{SE_{fN}},\rho_{S}⊗\rho_{E_{fN}})$. Their behaviour is shown in Figure 1b,c, respectively, as a function of the parameter *c*, which fixes the initial environmental state and therefore the model. As follows from their expressions given in Equations (12) and (14), their determination relies on knowledge of the eigenvalues of $\rho_{SE_{fN}}$, $\rho_{S}⊗\rho_{E_{fN}}$ and their average. In turn, these operators depend on the chosen initial state for the system that we have taken to be the pure state $\rho_{S}\left(0\right)=\left|+\rangle \langle +\right|$, initially exhibiting the maximum amount of coherence so as to better put into evidence the role of the environment.

For the case c=0, one immediately sees from Equation (11) that the environment is left unchanged so that it remains in the maximally mixed state. The eigenvalues of the relevant states can be shown to be

(15) $$\begin{array}{ccc} \rho_{SE_{fN}}\left(s\right) & \to & \frac{1}{2^{fN+1}}\left(1\pm \cos^{N-fN}\left(2gs\right)\right)\\ \rho_{S}\left(s\right) & \to & \frac{1}{2}\left(1\pm \cos^{N}\left(2gs\right)\right)\\ \rho_{E_{fN}}\left(s\right) & \to & \frac{1}{2^{fN}}\\ \frac{\rho_{SE_{fN}}\left(s\right)+\rho_{S}\left(s\right)⊗\rho_{E_{fN}}\left(s\right)}{2} & \to & \frac{1}{2^{fN+1}}\left(1\pm \frac{1}{2}\left|\cos^{N}\left(2gs\right)+\cos^{(1-f)N}\left(2gs\right)e^{i2g\left(\sum_{k=1}^{fN}m_{k}\right)s}\right|\right) \end{array}$$

where the eigenvalues for $\rho_{SE_{fN}}\left(s\right)$ and $\rho_{E_{fN}}\left(s\right)$ are $2^{fN}$ degenerate, while the numbers $\left\{m_{k}\right\}$ belong to {−1,1} and their value is determined by the associated eigenvector. In terms of these expressions, exploiting the fact that the von Neumann entropy of a state only depends on its eigenvalues,

(16) $$\begin{array}{ccc} S(\rho ) & = & -\sum_{i}\rho_{i}\log\rho_{i}, \end{array}$$

one can analytically determine the relevant expressions for the correlations.

An arbitrary value of the coherences in the initial environmental state calls for a numerical evaluation, whose results are shown in Figure 1b,c at time gs=π/4, when the system has fully decohered, as can be seen from Equations (8) and (9), thus losing its initial information content. It immediately appears, independently of the chosen correlation quantifier, that for c=12, i.e., initially pure environmental units, the system shares an equal amount of correlations with any small fraction of the environment, corresponding to a plateau in the fraction dependence of the correlation quantifiers. This feature is interpreted in the literature as quantum Darwinism [3], namely, a redundant storing of information about the system in different portions of the environment, allowing for a notion of objectivity, in the sense that the same information can be retrieved by different observers accessing distinct parts of the environment. It is to be stressed that the mutual information provides the standard choice of a correlation quantifier in this framework, though others were also considered [40,44]. This notion of objectivity is not uncontroversial; see [7] for a critical discussion and further developments. The formation of the plateau is slowed down with decreasing *c*, while for c=0, such that the environmental units are maximally mixed, correlations are only established between the system and the environment as a whole. This behaviour, namely, the gradual washing out of Darwinism in the dependence on the state of the environmental units, was already observed in [50], where the von Neumann entropy of the units was used as the figure of merit, which is a monotonic function of the coherence; see also [39,51].

To exemplify the distribution of correlations for the case c=0, let us write the overall state Equation (11) for the case of two environmental qubits, thus obtaining

(17) $$\rho_{SE}\left(s\right)=\frac{1}{8}\left(\begin{array}{cc} 1 & e^{-i4gs}\\ e^{i4gs} & 1 \end{array}\right)⊗\left(\begin{array}{cccc} 1 & 0 & 0 & 0\\ 0 & 0 & 0 & 0\\ 0 & 0 & 0 & 0\\ 0 & 0 & 0 & 0 \end{array}\right)+\frac{1}{8}\left(\begin{array}{cc} 1 & 1\\ 1 & 1 \end{array}\right)⊗\left(\begin{array}{cccc} 0 & 0 & 0 & 0\\ 0 & 1 & 0 & 0\\ 0 & 0 & 1 & 0\\ 0 & 0 & 0 & 0 \end{array}\right)\;\;\;\;\;\;\;\;\;\;\;\;\;\;\;\;+\frac{1}{8}\left(\begin{array}{cc} 1 & e^{i4gs}\\ e^{-i4gs} & 1 \end{array}\right)⊗\left(\begin{array}{cccc} 0 & 0 & 0 & 0\\ 0 & 0 & 0 & 0\\ 0 & 0 & 0 & 0\\ 0 & 0 & 0 & 1 \end{array}\right),$$

namely, a classically correlated state, apart from multiples of gs=π/2, when the factorised initial state is recovered due to periodicity. Tracing out all but one of the environmental qubits, we obtain

(18) $$\rho_{SE_{1}}\left(s\right)=\frac{1}{8}\left(\begin{array}{cc} 2 & 1+e^{-i4gs}\\ 1+e^{i4gs} & 2 \end{array}\right)⊗\left(\begin{array}{cc} 1 & 0\\ 0 & 0 \end{array}\right)+\frac{1}{8}\left(\begin{array}{cc} 2 & 1+e^{i4gs}\\ 1+e^{-i4gs} & 2 \end{array}\right)⊗\left(\begin{array}{cc} 0 & 0\\ 0 & 1 \end{array}\right),$$

which is immediately seen to be factorized for gs=π/4, i.e., when the system has fully decohered. Accordingly, the information about the reduced system is stored then solely in the global correlations between the system and the environment, and all partial fractions of the environment are not correlated with the reduced system (note that the reduced density matrix of the environment is always maximally mixed). This is exactly the behaviour appearing in Figure 1b,c.

## 4. Information Backflow

We now want to analyse the features of the different models in the framework of non-Markovianity, which is the study of memory effects in a quantum setting. In this respect, we will make reference to an approach to quantum non-Markovianity focusing on features of the reduced dynamics [8,9], at variance with viewpoints which more closely mimic the classical definition of a non-Markovian process, referring to joint probability distributions [52], thus involving information on intermediate steps necessary in order to extract information from a quantum system [53]. Given that the considered definition of non-Markovian dynamics only involves the reduced dynamics, the whole class of considered initial conditions performs in exactly the same way. Nevertheless, the definition to be considered relies on the information exchange between the system and environment, which manifests differently in the various models.

Let us first briefly formalise the pioneering approach to the non-Markovianity of a quantum dynamics introduced in [54,55]. The basic idea is to consider the evolution in time of the distinguishability between two system states, associating to a non-monotonicity in the time of this quantity the definition of non-Markovian dynamics. The motivation behind this definition is that revivals of distinguishability can be unambiguously associated to information backflow from external degrees of freedom to the system. This approach was initially formulated in terms of the trace distance [9], but is actually amenable to the use of other distinguishability quantifiers, in particular, entropic ones, which come closer to the present treatment focused on the spreading of correlations, as shown in [45,46].

**Figure 2:**
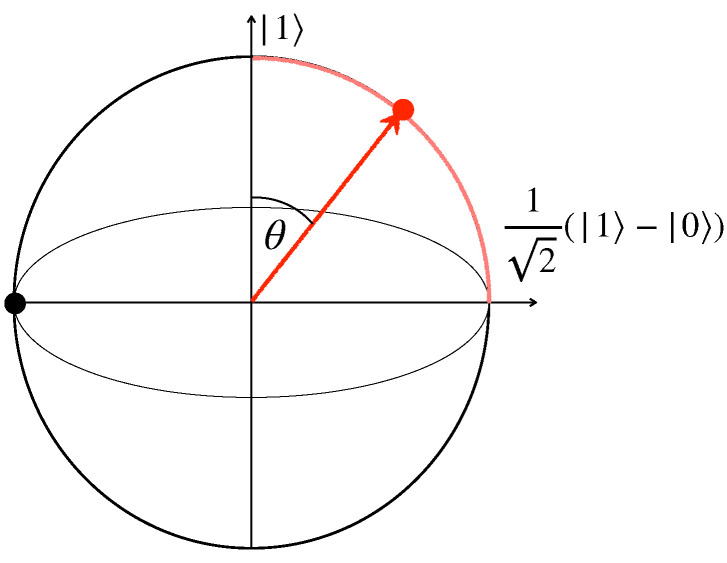
Bloch sphere representation of the considered pair of initial system states. One state is fixed to be the equatorial plus state $|+\rangle =\left(1/\sqrt{2}\right)(|0\rangle +\left|1\rangle \right)$ (black dot), while the other element of the pair belongs to the maximum circle and is characterised by the angle θ (red dot). For θ=π/2 it becomes the minus state $|-\rangle =\left(1/\sqrt{2}\right)(|0\rangle -\left|1\rangle \right)$ and one recovers an orthogonal pair of initial states. For θ=0, it corresponds to the up state |1〉.

The key quantity to be considered is therefore the distinguishability of two system states evolved from two distinct initial conditions, namely

(19) $$\sqrt{J(\rho_{S}^{1}\left(s\right),\rho_{S}^{2}\left(s\right))},$$

where, as discussed, we used as the distinguishability quantifier the QJSD12 as defined in Equation (12). The QJSD12 is a contraction with respect to the action of any positive trace-preserving map so that $\sqrt{J(\rho_{S}^{1}\left(s\right),\rho_{S}^{2}\left(s\right))}$ is monotonically decreasing in the case of a positive divisible evolution. For more general dynamics, this quantity can show revivals in time, pointing to the existence of memory effects. In particular, the revivals from the value at a time *s* to a value at a later time *t* can be upper bounded according to

(20) $$\begin{array}{c} \sqrt{J(\rho_{S}^{1}\left(t\right),\rho_{S}^{2}\left(t\right))}-\sqrt{J(\rho_{S}^{1}\left(s\right),\rho_{S}^{2}\left(s\right))}\\ ⩽\sqrt{J(\rho_{E}^{1}\left(s\right),\rho_{E}^{2}\left(s\right))}+\sqrt{J(\rho_{SE}^{1}\left(s\right),\rho_{S}^{1}\left(s\right)⊗\rho_{E}^{1}\left(s\right))}+\sqrt{J(\rho_{SE}^{2}\left(s\right),\rho_{S}^{2}\left(s\right)⊗\rho_{E}^{2}\left(s\right))}, \end{array}$$

where $\rho_{E}^{1,2}\left(s\right)$ denote the time-evolved environmental states corresponding to the initial condition $\rho_{S}^{1,2}\left(0\right)$, while $\rho_{E}^{1}\left(0\right)=\rho_{E}^{2}\left(0\right)$ is determined as above by fixing the model of interest. Given that all three contributions at the r.h.s. are zero if and only if their arguments are equal, this bound has a clear physical meaning: non-Markovianity as described by revivals in the distinguishability of system states can only take place if correlations have been established between the system and environment, which is captured by the last two terms on the r.h.s of Equation (20) and/or different initial system states have affected, in a different way, the state of the environment, captured by the first term on the r.h.s. of Equation (20). In both cases, some information is stored in degrees of freedom external with respect to the system. The revivals do depend, in general, on the choice of initial system states so that it is natural to consider initial pairs that can be perfectly distinguished, namely, orthogonal states. In our case, given the previously considered choice $\rho_{S}^{1}\left(0\right)=\left|+\rangle \langle +\right|$, this would amount to considering $\rho_{S}^{2}\left(0\right)=\left|-\rangle \langle -\right|$. However, one immediately realises that in analogy to the fact that the reduced system dynamics is only affected by the diagonal matrix elements of the environmental qubits, also the dynamics of the environmental states only depends on the diagonal elements of $\rho_{S}^{1,2}\left(0\right)$ in the $\sigma_{z}$ basis. This would automatically imply the vanishing of the first term at the r.h.s. of Equation (20). We therefore consider a more general pair of initial states, namely $\rho_{S}^{1}\left(0\right)=\left|+\rangle \langle +\right|$ and $\rho_{S}^{2}\left(0\right)=\left|\theta \rangle \langle \theta \right|$, with |θ〉=cos(θ/2)|1〉−sin(θ/2)|0〉, as depicted in Figure 2.

We now want to explore the behaviour of these bounds for the different considered microscopic models, investigating, in particular, what happens when only partial information on the environment can be obtained.

### 4.1. Model Dependence of Bounds on Distinguishability Revivals

**Figure 3:**
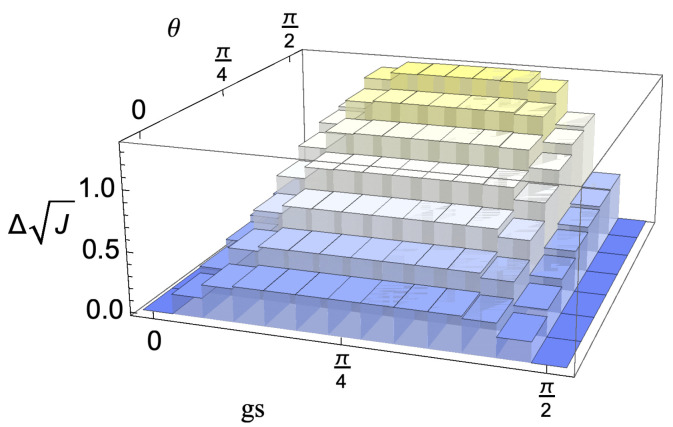
Plot of the l.h.s. of Equation (20) showing the revivals of the QJSD12 as a function of time and choice of initial system states. The reference time gt is fixed to be π/2, i.e., after one full period of the evolution, while gs sweeps from 0 to π/2. The initial pair of system states is given by $\rho_{S}^{1}\left(0\right)=\left|+\rangle \langle +\right|$ and $\rho_{S}^{2}\left(0\right)=\left|\theta \rangle \langle \theta \right|$, as shown in Figure 2, with θ ranging from 0 to π/2, corresponding to the case of an orthogonal pair and maximizing the revivals.

**Figure 4:**
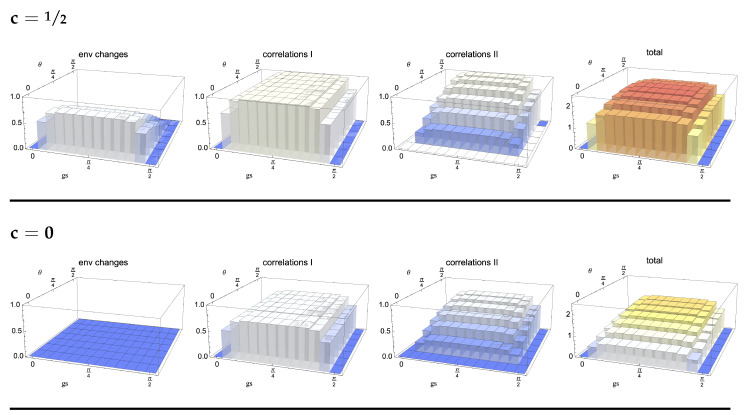
Plot of the different contributions at the r.h.s. of Equation (20), together with their sum, all quantified via the QJSD12. They are considered as a function of running time gs and initial pair of states fixed by the angle θ. The first row corresponds, as indicated, to the model determined by c=12, the second to c=0. For c=12, the environmental units have the maximum amount of coherence, while for c=0, they start in a maximally mixed state and the reduced environmental state remains unchanged, so that one of the contributions is equal to zero.

We first analyse the behaviour in time of the bounds, exploring their dependence on the considered model. In particular, we investigate the models arising for the choices c=0 and c=12. We recall that the non-Markovianity only depends on the behaviour of the reduced state of the system so that it is exactly the same for all values of *c*. The revivals of distinguishability for the whole class of initial conditions, expressed by means of the QJSD12, namely the l.h.s. of Equation (20), are shown in Figure 3 as a function of the rescaled time and the choice of initial system states. As expected, the highest revivals take place for orthogonal initial states, corresponding to θ=π/2. The periodicity of the dynamics, due to the uniform coupling, is also apparent. In Figure 4, we show the behaviour of the contributions at the r.h.s. of the bound, which provide information on degrees of freedom, external with respect to the system, so that they are indeed model dependent. We plot the contributions due to established correlations, starting from the initial conditions $\rho_{S}^{1}\left(0\right)$ and $\rho_{S}^{2}\left(0\right)$, respectively, together with the distinguishability of the correspondingly evolved environmental states $\rho_{E}^{1}\left(s\right)$ and $\rho_{E}^{2}\left(s\right)$, as well as the sum of the three terms, which determines the overall tightness of the bound, Equation (20). The first row shows the result for the model corresponding to c=12, in which Darwinism appears, the second for c=0. We see that in the first case, the upper bound is significantly less tight. The main reason is that for c=0, the environmental state does not evolve so that one of the contributions is always zero, while in the other model, changes of the environmental dynamics take place for all choices of the θ parameter different from π/2, corresponding as discussed above to $\rho_{S}^{2}\left(0\right)=\left|-\rangle \langle -\right|$. The correlations between the second reduced system state and its environment, the only θ-dependent ones, are strongly affected by the parameter fixing this second initial system state but in the opposite manner with larger θ leading to more pronounced correlations. As a result, the upper bound is only weakly θ dependent. Interestingly, the maximum of the upper bound as a function of θ does not correspond to the maximum of the bounded quantity, namely the l.h.s. of Equation (20), shown in Figure 3. In all cases, the dominant contribution is given by the established correlations.

### 4.2. Fraction Dependence of Bounds on Distinguishability Revivals

**Figure 5:**
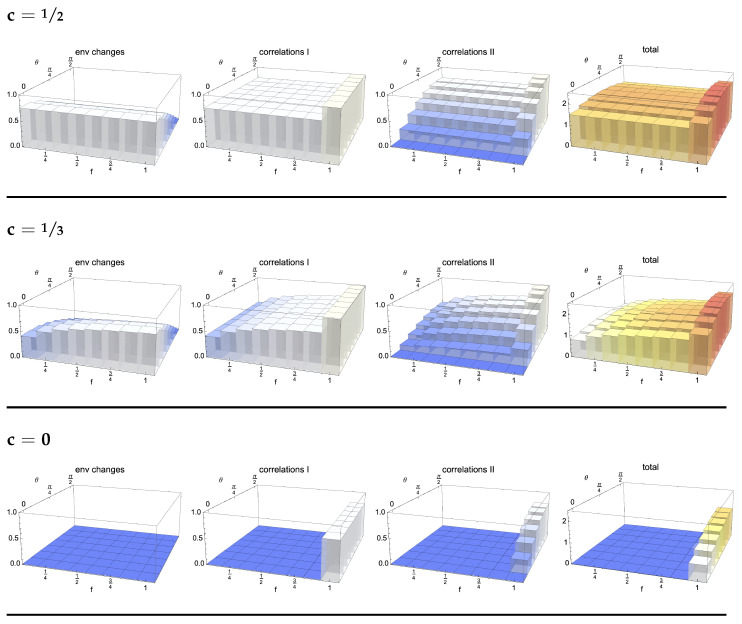
Plot of the different contributions at the r.h.s. of Equation (20), together with their sum, evaluated for the case in which the total state is replaced by a marginal obtained by tracing out some environmental qubits, so that only a fraction f is considered. Additionally, in this case, all quantities are expressed via the QJSD12. They are considered a function of fraction f and initial pair of states determined by the angle θ for a fixed time gs=π/4. The first row corresponds, as indicated, to the model determined by c=12, the second to c=13 and the third to c=0. For c=12, plateaus as a function of f are clearly observed, replaced for c=13 by a weak dependence. For c=0, a non-zero value is only obtained when including the whole environment since tracing over any environmental units leads to a factorised state.

**Figure 6:**
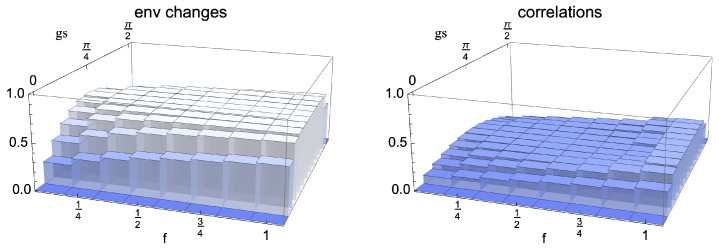
Behaviour of environmental changes and correlations for the model with c=12 plotted as a function of both fraction f and time gs. It immediately appears that a plateau as a function of f only takes place for the time gs=π/4, corresponding to full decoherence of the system.

In order to understand the role of the spreading of information for the description of non-Markovianity in the different models, we study the behaviour of the quantities at the r.h.s. of Equation (20) when replacing the environment with a smaller one, given by a fraction of the original set of degrees of freedom. To this aim, we fix a reference time taken to be gs=π/4, corresponding to full decoherence of the reduced system, when quantum Darwinism is typically observed. Since the partial trace is a completely positive trace preserving map, each contribution gets smaller due to contractivity of the QJSD12 under such maps, a feature shared by all distinguishability quantifiers considered for the description of memory effects. The inequality in Equation (20) is therefore no longer required to hold true since we are lowering the r.h.s. without affecting the l.h.s. The model corresponding to c=0, see last row of Figure 5, is very special in this respect, as no information whatsoever is stored in any fraction of the environment smaller than the total environment so that the bound is immediately violated. For all choices of initial reduced states, correlations are built solely with the total environmental state, while by tracing out any number of environmental qubits, one obtains a factorised state. Additionally, the maximally mixed environmental state is invariant during the evolution for all choices of reduced initial state, a property which is obviously preserved by taking into account only some fraction of environmental degrees of freedom. On the other hand, in the model obtained for c=12, such that the initial environmental states are pure, as shown in the first row of Figure 5, the difference in environmental states does not depend on the fraction of the environment we are taking into account. To see the origin of this behaviour, we come back to Equation (11) evaluated at time gs=π/4 for c=12, which, upon taking the partial trace with respect to system and a fraction f of the environment, leads to the state

(21) $$\rho_{E_{fN}}\left(\pi /\left(4g\right)\right)=\rho_{11}\left(0\right)\frac{1}{2^{fN}}\overset{fN}{\underset{k=1}{⨂}}\left(\begin{array}{cc} 1 & -i\\ i & 1 \end{array}\right)+\rho_{00}\left(0\right)\frac{1}{2^{fN}}\overset{fN}{\underset{k=1}{⨂}}\left(\begin{array}{cc} 1 & i\\ -i & 1 \end{array}\right),$$

whose only non-zero eigenvalues are $\rho_{11}\left(0\right)$ and $\rho_{00}\left(0\right)$ so that the difference in environmental states is not influenced by the number of environmental units taken into account. We stress that this is only true for gs=π/4, the point in time most relevant for the study of quantum Darwinism. This can be seen considering both the dependence on time and fraction as in Figure 6, where both environmental changes and correlations are considered. Remarkably, for this particular model, the occurrence of a plateau as a function of the environmental fraction is not only true for the changes in the environment, but also in the correlations and, consequently, in the sum of these three quantities providing the overall bound. In other words, the information exchange relevant for the onset of non-Markovianity only involves a small portion of the environment, so that the bound holds for any considered fraction. The appearance of these plateaus makes the dynamics indeed compatible with quantum Darwinism, even though it only provides a sufficient condition for the redundant spreading of information.

**Figure 7:**
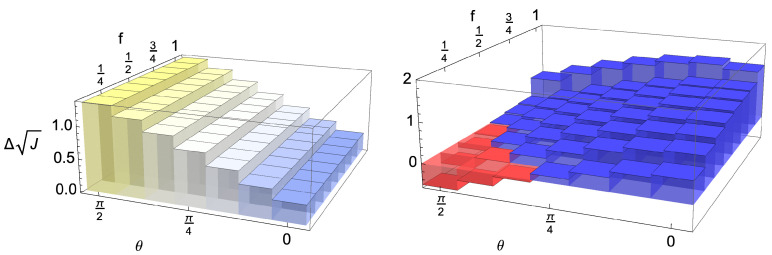
(**Left**) Plot of the l.h.s. of Equation (20) showing the revivals of the QJSD12 as a function of choice of initial system states for the fixed times gt=π/2 and gs=π/4, the latter corresponding to maximal decoherence of the system. The quantity inherently does not depend on f. (**Right**) The difference of the l.h.s. and sum of quantities on the r.h.s. of the inequality given by Equation (20) when taking into account only a fraction f of the environment, for c=13 (see Figure 5, last figure of the second row). For the values of environmental fraction f and angle θ (which determines the pair of initial system states) for which the difference is negative (red), the corresponding sum of environmental changes and correlations is no longer an upper bound for the revivals in the reduced dynamics.

The occurrence of a very weak dependence with respect to the stepwise inclusions of environmental degrees of freedom is not new; it was indeed already observed in a collisional framework [22]. It reflects the fact that given the size of the system, the correlation with a small portion of the environment is already sufficient to store the information necessary to lead to a revival in distinguishability of the system states. In the present framework, for c=12, we are faced with a true plateau, which reflects the pure Darwinistic behaviour exhibited by this model. To better clarify this behaviour, we have also plotted the same quantities for an intermediate choice of the mixing parameter c=13; see middle row of Figure 5. In this case, corresponding to a model in which Darwinism is partially washed out [50], a weak dependence on the size of the fraction can be observed, so that a larger part of the environment is necessary to recover the relevant information. For this model, the failure of the upper bound upon tracing out part of the environment can be observed for a small enough fraction and large values of the parameter θ; see Figure 7. While the amount of information flowing back to the open system does not depend on the fraction of the environment taken into account, the capability to trace it back to the established correlations between the system and a portion of the environment, as well as to the changes of the latter, requires now that a large enough portion is considered.

## 5. Conclusions

We have investigated the subtle role that system–environment correlations play in the characterisation of a given dynamics. Through a paradigmatic dephasing model with different initial conditions, which are particularly relevant in exploring the quantum Darwinism framework, we employed tools from the study of non-Markovianity to critically assess the role that these correlations play, revealing that while only a small amount of such correlations are needed for the onset of non-Markovianity, establishing the conditions for classical objectivity necessitates significantly more. Our results indicate that for most microscopical realisations of the reduced dynamics, one can fully capture the non-Markovian characteristics of a given evolution with access to only a small subset of the environmental degrees of freedom, while also revealing that whether the conditions for classical objectivity are satisfied or not is crucially dependent on the precise details of the microscopic model in question, rather than its non-Markovian nature.

## Data Availability

Not applicable.
